# Characterization of regulatory genes *Plhffp* and *Plpif1* involved in conidiation regulation in *Purpureocillium lavendulum*

**DOI:** 10.3389/fmicb.2024.1352989

**Published:** 2024-02-15

**Authors:** Yu Wei, Feng-Na Qi, Yan-Rui Xu, Ke-Qin Zhang, Jianping Xu, Yan-Ru Cao, Lian-Ming Liang

**Affiliations:** ^1^State Key Laboratory for Conservation and Utilization of Bio-Resources in Yunnan, Yunnan University, Kunming, China; ^2^Department of Biology, McMaster University, Hamilton, ON, Canada; ^3^College of Agriculture and Life Sciences, Kunming University, Kunming, China

**Keywords:** *Purpureocillium lavendulum*, *Agrobacterium tumefaciens* – mediated transformation, T-DNA random insertion mutant library, conidiation genes, biocontrol

## Abstract

*Purpureocillium lavendulum* is an important biocontrol agent against plant-parasitic nematodes, primarily infecting them with conidia. However, research on the regulatory genes and pathways involved in its conidiation is still limited. In this study, we employed *Agrobacterium tumefaciens*-mediated genetic transformation to generate 4,870 random T-DNA insertion mutants of *P. lavendulum*. Among these mutants, 131 strains exhibited abnormal conidiation, and further in-depth investigations were conducted on two strains (designated as #5-197 and #5-119) that showed significantly reduced conidiation. Through whole-genome re-sequencing and genome walking, we identified the T-DNA insertion sites in these strains and determined the corresponding genes affected by the insertions, namely *Plhffp* and *Plpif1*. Both genes were knocked out through homologous recombination, and phenotypic analysis revealed a significant difference in conidiation between the knockout strains and the wild-type strain (*ku80*). Upon complementation of the Δ*Plpif1* strain with the corresponding wildtype allele, conidiation was restored to a level comparable to *ku80*, providing further evidence of the involvement of this gene in conidiation regulation in *P. lavendulum*. The knockout of *Plhffp* or *Plpif1* reduced the antioxidant capacity of *P. lavendulum*, and the absence of *Plhffp* also resulted in decreased resistance to SDS, suggesting that this gene may be involved in the integrity of the cell wall. RT-qPCR showed that knockout of *Plhffp* or *Plpif1* altered expression levels of several known genes associated with conidiation. Additionally, the analysis of nematode infection assays with *Caenorhabditis elegans* indicated that the knockout of *Plhffp* and *Plpif1* indirectly reduced the pathogenicity of *P. lavendulum* towards the nematodes. The results demonstrate that *Agrobacterium tumefaciens* - mediated T-DNA insertion mutagenesis, gene knockout, and complementation can be highly effective for identifying functionally important genes in *P. lavendulum*.

## 1 Introduction

Plant-Parasitic Nematodes (PPNs) have a wide range of hosts, ranging from lower plants like mosses, ferns, and algae, to higher plants such as gymnosperms and angiosperms (Shao et al., 2023). Almost all plants are affected by plant-parasitic nematodes to varying degrees. PPNs pose a serious threat to numerous economically important crops worldwide (Rehman et al., 2016) and are a significant limiting factor for global food security (Montarry et al., 2021). In economically underdeveloped regions, due to a lack of relevant control measures, the damage caused by nematodes to food crops is more severe than in developed regions (Coyne et al., 2018).

**FIGURE 1:**
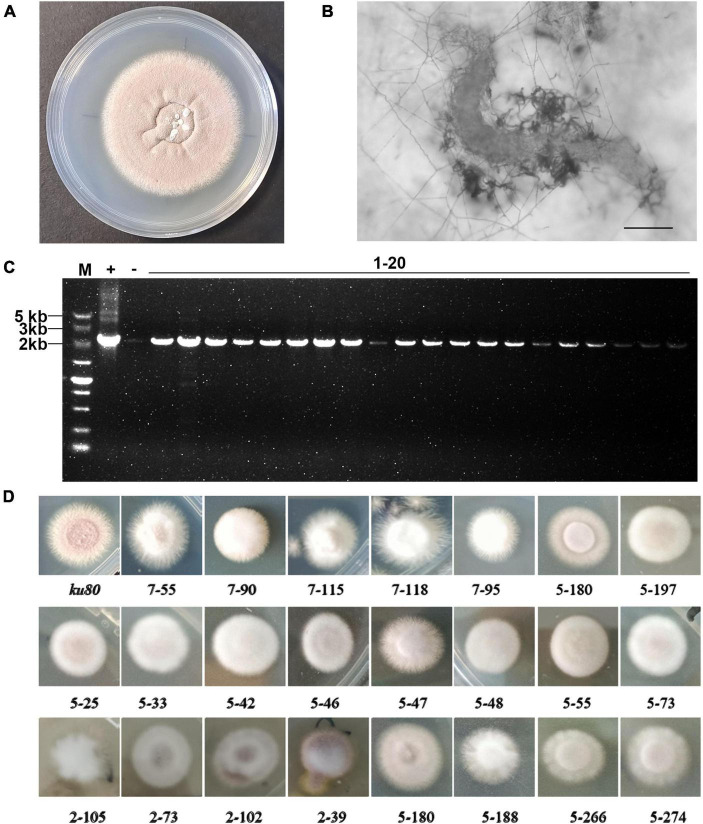
Construction and screening of the T-DNA random insertion library. **(A)** The purple-color colony of the *P. lavendulum ku80* strain. **(B)** A nematode infected by *P. lavendulum* (scale bar, 50 μm). **(C)** T-DNA insertion in the transformants was confirmed by PCR amplification of the *sur* gene. M, DNA marker. + PCR with the plasmid pPK2-sur-GFP as template. –, PCR with the *ku80* genomic DNA as template. **(D)** Colony morphology of *ku80* and conidiation defect strains.

Currently, the control and management of PPNs mainly involve agricultural practices, physical control, chemical control, biological control, and the use of plant-based pesticides (Li et al., 2015). Biological control typically involves the use of nematode predators, such as *Nematophagous* microorganisms, to control nematode populations. Some bacterial pathogens of nematodes produce toxic substances that can kill nematodes, while certain fungi have evolved specific predatory structures to capture and feed on nematodes (Liang et al., 2019). *Purpureocillium lavendulum* is an important bio-control fungus against nematodes (Figure 1A). Compared to its sister species, *P. lilacinum*, its inability of growth at temperatures above 35°C makes it safe for humans and other mammals (Perdomo et al., 2013).

Conidiation, the formation of asexual conidia, is a crucial process employed by *P. lavendulum* for infecting nematodes, with conidia serving as the infectious propagule in this interaction (Figure 1B). However, research on conidiation regulatory genes and mechanisms in *P. lavendulum* is still limited. Our previous studies identified three genes involved in *P. lavendulum* conidiation: *PlbrlA*, *PlabaA*, and *PlwetA* (Chen et al., 2020). These genes respectively control the early, middle, and late stages of asexual development. Deletion of *PlbrlA* completely inhibits the formation of conidia, resulting in the production of conidiophores without conidial formation. *PlabaA* determines the differentiation of conidia on conidiophores. *PlwetA* affects various phenotypes related to conidial maturation, including conidial detachment from conidiophores, cell wall thickening, vacuole formation, trehalose production, and heat shock tolerance, among others (Chen et al., 2020). In addition, conidiation of *P. lavendulum* was also found be co-regulated by nitrogen sources and histone H3K14 acetylation (Tang et al., 2023). While the core regulatory genes and a few upstream genes have been defined, the genes that they interact with to impact conidiation remain largely unknown.

Mutant library construction and screening is an effective approach for studying unknown genes and their biological functions. In recent years, *Agrobacterium*-mediated genetic transformation to construct T-DNA random insertion mutant libraries has been widely used to study filamentous fungi, such as *Aspergillus terreus*, *Colletotrichum higginsianum*, and *Trichoderma reesei* (Yao Hua et al., 2007; Wang et al., 2014; Tanaka et al., 2023). In this study, we utilized Agrobacterium-mediated genetic transformation to construct a T-DNA random insertion mutant library of *P. lavendulum.* Screening of the library led to the identification of two conidiation mutants that we further characterized using targeted gene knockout and complementation.

## 2 Materials and methods

### 2.1 Strains and plasmids

*Purpureocillium lavendulum ku80*, a *ku80* gene knockout strain with a high homologous recombination rate, was used as the parental strain for mutant library construction and gene knockout (Liu et al., 2019). *E. coli* DH5α competent cells were used to construct knockout plasmids. *Agrobacterium tumefaciens* AGL-1 was used for fungal transduction. The pPK2-sur-GFP plasmid was kindly provided by Professor Fang Weiguo from Zhejiang University, and it was used for constructing the mutant library of *P. lavendulum* (Xu et al., 2014).

### 2.2 Agrobacterium-mediated genetic transformation of *P. lavendulum*

Fungal transformation followed a previously described method (Zhuang et al., 2023). Briefly, the pPK2-sur-GFP plasmid was transferred into *Agrobacterium tumefaciens* AGL-1 competent cells using the liquid nitrogen freezing method described in the reference manual. The *Agrobacterium* cells harboring the pPK2-sur-GFP plasmid were inoculated into LB liquid medium and incubated on a shaker at 28°C until reaching an optical density (OD) of 0.45. At this point, the *Agrobacterium* culture was mixed with *P. lavendulum* conidia at a 1:1 ratio, avoiding light exposure. The mixture is evenly spread on IM medium supplemented with acetosyringone and covered with a microporous filter membrane. The plates were incubated inverted at 22°C for 48 hours. Afterward, the microporous filter membrane was transferred to M-100 medium (with 50 μg/ml chlorsulfuron) and incubated at 28°C for 4–6 days until transformants grow out. A 20 mutant strains were randomly selected for confirm of transformation analysis by PCR amplifying sulfonylurea resistance gene (*sur*) with the primers surF/surR (Supplementary Table 1).

### 2.3 Screening of the mutant library of *P. lavendulum*

The obtained transformants were subjected to screening by inoculating them on PDA agar plates and incubation at 28°C for 4 days and comparing their colony morphologies. The conidia of *P. lavendulum* are purple, and the wild-type strain (*ku80*) exhibits a high conidiation rate, resulting in colonies with a purple color. Therefore, to identify transformants with reduced conidiation, the ones showed pale purple or white colonies were selected.

### 2.4 Identification of the T-DNA insertion sites

Two strategies were utilized for identifying the T-DNA insertion sites, genome walking and genome re-sequencing, in the target strains with reduced conidiation. In the genome walking method, the Takara Genome Walking Kit (Takara, Catalog Number: 6108) was used for three rounds of PCR amplification using primers in the kit and designed according to the T-DNA sequence (Supplementary Table 1). The obtained bands were sequenced and compared using Local BLAST in the software BioEdit to identify fragments that match the *P. lavendulum* genome (Hall, 1999). By aligning these fragments with the T-DNA sequence, potential insertion sites of the T-DNA were identified.

Selected strains with reduced conidiation were subjected to whole-genome re-sequencing. The reads partially mapped to T-DNA were mapped to the reference genome and thus the potential insertion position was identified. After that, primers were designed for PCR reactions to validate the predicted T-DNA insertion site and determine the precise insertion point in each mutant (primers were listed in Supplementary Table 1).

### 2.5 Gene knockout and complementation

Gene knockouts were carried out by homologous recombination. The homologous arms were amplified with primers in Supplementary Table 1, and then inserted into the plasmid ppk2-sur-gfp by infusion method, at the *Xba*I/Bg1II and *Spe*I/*Eco*RV sites, respectively. To construct the gene complementation plasmid of *Plpif1*, the complete gene, including the open reading frame (ORF) region, along with 2kb upstream and 0.5 kb downstream regions, was amplified by PCR and then ligated to the ppk2-bml-GFP plasmid [reported previously(Liu et al., 2019)]. The plasmid was then transformed into the fungus using Agrobacterium-mediated transformation method. Transformants grew on screening plates were then confirmed by PCR (primers were listed in Supplementary Table 1).

### 2.6 Morphology observation and comparison

After obtaining the knockout strains as well as the complementation strain, they were cultured on regular solid media MM, TG, and CMA, as well as on stress-inducing media (MM with various concentrations of SDS, NaCl, or H_2_O_2_). The growth rates and conidia production were determined and compared with the *ku80* strain.

### 2.7 Real-time quantitative PCR

The expression levels of conidiation regulation genes previously reported (Chen et al., 2020), such as *PlfluG*, *PlflbA*, *PlbrlA*, *et.al.*, were selected to measure their expression levels with real-time quantitative PCR. RNA was extracted from mycelia of *ku80* and the two mutant strains using RaPure Total RNA Micro Kit (Magen, Guangzhou, China). Real-time quantitative PCR were carried out by SYBR-Green method with KAPA SYBR FAST qPCR Kit (Roche) on the LightCycler-^®^ 480 Instrument II (Roche). The *actin* gene was used as an internal reference. The primers used were listed in Supplementary Table 1.

## 3 Results

### 3.1 Establishment of a random T-DNA insertion mutant library in *P. lavendulum* using ATMT

Using the *P. lavendulum ku80* strain as the parental strain, genetic transformation mediated by *Agrobacterium tumefaciens* was employed to introduce the T-DNA of the plasmid pPK2-sur-GFP into the *ku80* genome, constructing a random T-DNA insertion mutant library of *P. lavendulum*. After multiple batches of genetic transformation, a total of 4,870 mutant strains were obtained. To evaluate the efficiency of T-DNA insertion into the *P. lavendulum* genome, 20 mutant strains were randomly selected for analysis. PCR results (The target band size amplified by the designed primers was 2145 bp) showed that all 20 randomly selected mutant strains had T-DNA insertions (Figure 1C). This indicated that the success rate of using Agrobacterium-mediated transformation in *P. lavendulum* was relatively high.

### 3.2 Identification, screening, and preservation of the random T-DNA insertion mutant library in *P. lavendulum*

A total of 2650 transformants were screened for defect in conidiation. Among them, 131 strains showed reduced conidiation and increased white mycelium production. 37 strains had colony diameters significantly larger or smaller than the parental *ku80* strain when grown on the same culture medium. The colonies of some representative strains are shown in (Figure 1D). From the figure, we can see that the colony of *ku80* was purple on PDA medium, with a powdery appearance due to abundant conidia production. Most of the screened mutants exhibited reduced conidiation, increased white or pale purple mycelium, irregular colony morphology with serrated edges or uniform extension of mycelium, while some colonies were round and full, fluffy, and exhibited a delicate mycelial appearance instead of a powdery appearance. The obtained mutant strains were classified based on phenotypes to establish a random mutant library of *P. lavendulum*.

Large-scale preservation of strains is crucial for the repeated utilization of the mutant library. The following preservation methods for mutant strains of *P. lavendulum* were selected and tested: (1) 25% glycerol at −80°C, (2) 50% glycerol at −80°C, and (3) deionized water at 4°C. To determine the best preservation method among the three mentioned above, 1 mL (1 × 10^8^ml^–1^) conidia of the *ku80* strain was preserved in each cryovial using the three preservation methods. One month later, three cryovials were taken out from each preservation method, and 100 μL of the thawed suspension was uniformly spread on MM medium. The results after 3 days of incubation is shown in Supplementary Figure 1A. It can be seen from the figure that the conidia preserved in 50% glycerol showed significantly slower germination and growth rates compared to the other two methods after one month of preservation. After six months, the same procedure was repeated with three cryovials from each preservation method, and the results are shown in Supplementary Figure 1B. It can be observed that conidia preserved using all three methods could germinate normally after six months, but the conidia preserved in 50% glycerol still exhibited slower germination and growth rates compared to the other two methods. Based on these results, we selected 25% glycerol at -80°C for preserving the mutant strains.

### 3.3 Identification of T-DNA insertion sites and affected genes

The T-DNA insertion site in strain #5-197 was identified in the gene contig1.1577 of *P. lavendulum* by whole-genome re-sequencing and was further confirmed through PCR. Through sequence BLAST in GenBank, its possible function was identified as an α, β hydrolase folds family protein (VFPFJ_05724) (Sequence ID: XM-018322804.1). This gene was named *Plhffp*.

The T-DNA insert site of strain #5-119 was identified by genome walking method. After sequencing and alignment, the PCR product was found to be 1388 bp in length. Among these, 834 bp matched the contig9 of the *P. lavendulum* genome, while 544 bp matched the T-DNA sequence. After PCR verification, the gene affected by the T-DNA insertion site was finally determined. Through Blast against the GenBank, its possible function was identified as ATP-dependent DNA helicase PIF1 (partial mRNA of PIF1) (VFPFJ_11645) (Sequence ID: XM-018328712.1). Therefore, this gene was named *Plpif1*.

### 3.4 The deletion of the *Plhffp* gene reduces conidiation and weakens cell wall synthesis and antioxidant capacity

**FIGURE 2:**
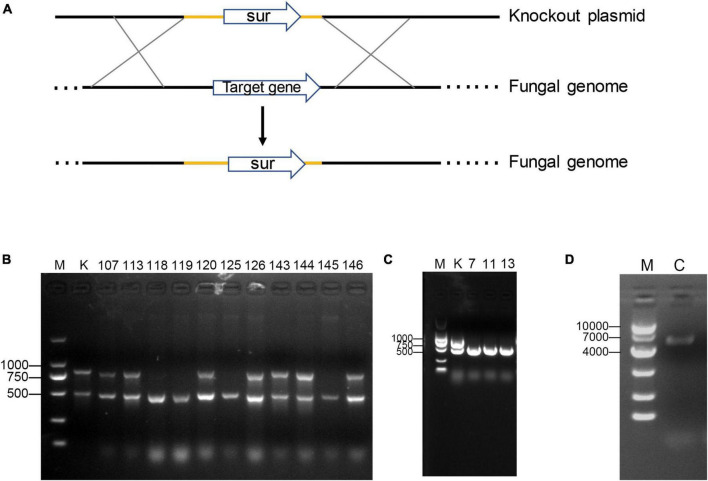
Gene knockout and complementation. **(A)** The Pattern diagram of homologous recombination knockout genes. **(B)** PCR screening of *Plhffp* mutants by amplifying the *Plhffp* gene (the upper band) and the *beta-tubulin* gene (the lower band). K, *ku80*. **(C)** PCR screening of *Plpif1* mutants by amplifying the *Plpif1* gene (the upper band) and the beta-tubulin gene (the lower band). K, *ku80*. **(D)** PCR confirmation of the complementation of *Plpif1* gene.

**FIGURE 3:**
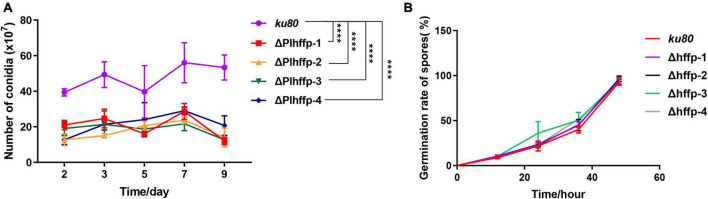
Comparison of conidiation **(A)** and conidia **(B)** germination of *ku80* and the Δ*Plhffp* strains. ****, *p* < 0.0001.

To determine the function of the two genes, we performed targeted gene knockout and complementation experiments. Unfortunately, *Plhffp* could not be complemented due to its high GC content, which made PCR amplification impossible (Figure 2). The growth rates of Δ*Plhffp* on MM, TG, and CMA media were not significantly different from the *ku80* strain (Supplementary Figure 2). However, the conidiation yield of Δ*Plhffp* was significantly decreased compared to *ku80* on the MM medium (Figure 3A). The germination rate of the mutant was similar to the *ku80* strain when cultured in MM medium (Figure 3B). This indicates that the knockout of the *Plhffp* gene affected conidiation without affecting the normal growth of the strain. On high osmolarity media, the growth rate of Δ*Plhffp* was not affected (Supplementary Figures 3A, C, E), indicating that the deletion of this gene does not impact the strain’s ability to resist high osmolarity. However, on media containing a high concentration of NaCl (0.3M), although the growth rate of Δ*Plhffp* was similar to *ku80*, the conidiation yield decreased significantly compared to *ku80* (Supplementary Figure 3F). Both growth and conidiation of the Δ*Plhffp* mutant were lower than that of the *ku80* strain when growing on MM media with 0.01–0.03% SDS, although not all of the differences were statistically significant (Supplementary Figure 4). High concentration of H_2_O_2_ also affected the growth and conidia of the Δ*Plhffp* mutant. The conidia production was significantly lower in the mutant than in *ku80* on 2.5 and 5 mM H_2_O_2_ (Supplementary Figure 5). It is speculated that deletion of the *Plhffp* gene may affect the integrity of the strain’s cell wall and its antioxidant capacity.

### 3.5 The deletion of the *Plpif1* gene reduces conidiation and weakens antioxidant capacity

**FIGURE 4:**
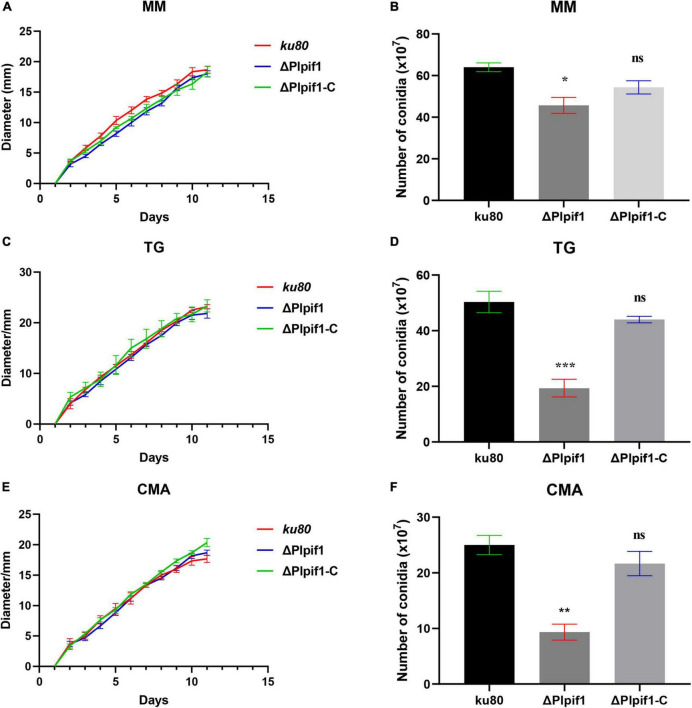
Comparison of growth and conidiation of *ku80*, the Δ*Plpif1* and Δ*Plpif1-C* strains on different media. **(A,B)** MM media; **(C,D)** TG media; **(E,F)** CMA media. Δ*Plpif1-C*, the complementary strain of ΔPlpif1.*, *p* < 0.05; **, *p* < 0.01; ***, *p* < 0.001; ns, not statistically significant.

**FIGURE 5:**
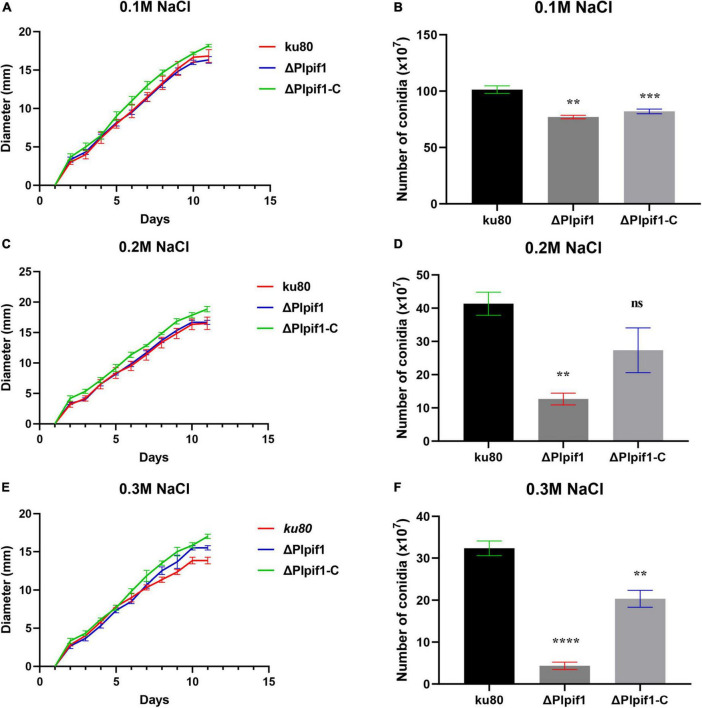
Comparison of growth and conidiation of *ku80*, the Δ*Plpif1* and Δ*Plpif1-C* strains on high osmotic media. **(A,B)** media with 0.1 M NaCl; **(C,D)** media with 0.2 M NaCl; **(E,F)** media with 0.1 M NaCl. **, *p* < 0.01; ***, *p* < 0.001; ****, *p* < 0.0001; ns, not statistically significant.

**FIGURE 6:**
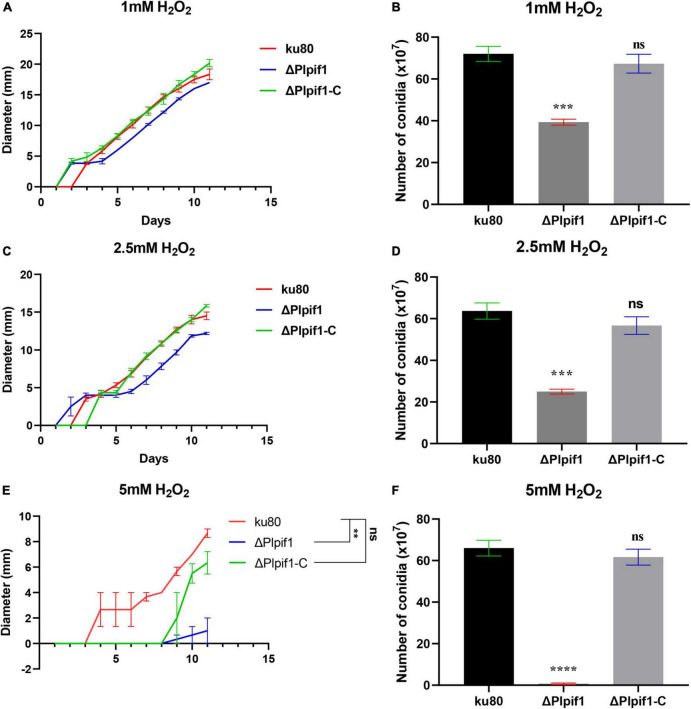
Comparison of growth and conidiation of *ku80*, the Δ*Plpif1* and Δ*Plpif1-C* strains on H_2_O_2_ containing media. **(A,B)** media with 1 mM H_2_O_2_; **(C,D)** media with 2.5 mM H_2_O_2_; **(E,F)** media with 5 mM H_2_O_2_. **, *p* < 0.01; ***, *p* < 0.001; ****, *p* < 0.0001; ns, not statistically significant.

The growth rates of Δ*Plpif1* and the complementary strain Δ*Plpif1-C* on MM, TG, and CMA media were not significantly different from the *ku80* strain (Figure 4). This indicates that the knockout of the Plpif1 gene does not affect the normal growth of the strain. However, it is worth noting that deletion of the Plpif1 gene significantly decreased conidiation on MM, TG, and CMA media compared to *ku80*. In addition, after complementation of the *Plpif1* gene, the conidiation yield was restored and showed no significant difference compared to *ku80* (Figure 4). This indicates that the knockout of the Plpif1 gene affects conidiation without impacting the normal growth of the strain. On high osmolarity media (with 0.1–0.3 M NaCl), the growth rates of Δ*Plpif1* and Δ*Plpif1-C* were consistent with *ku80*, showing no significant differences. However, the conidiation yield of Δ*Plpif1* was significantly lower than the control, while the conidiation yield of Δ*Plpif1-C* was restored to a level similar to *ku80* (Figure 5). Similar to Δ*Plhffp*, on H_2_O_2_ media, when the H_2_O_2_ concentration were 1 and 2.5 mM, Δ*Plpif1* strain grew similar to the *ku80* and the complementation strain, while conidiation was significantly decreased. When grew on 5 mM H_2_O_2_ medium, the Δ*Plpif1* mutant strain could hardly grow. However, after complementation of the *Plpif1* gene, the growth rate no longer showed a significant difference compared to *ku80* (Figure 6). This indicates that the deletion of the *Plpif1* gene, like the deletion of the *Plhffp* gene, reduces the antioxidant capacity of the fungus.

### 3.6 Deletion of the *Plhffp* and *Plpif1* genes affects the expression of key conidiation-related genes

Our previous study showed nine genes, *PlabaA*, *PlbrlA*, *PlfadA*, *PlflbA*, *PlflbC*, *PlflbD*, *PlfluG*, *PlpkaA*, and *PlwetA* to be associated with conidiation in *P. lavendulum* (Chen et al., 2020). Among them, three *PlbrlA*, *PlabaA*, *PlwetA* represented a central regulatory pathway for conidiation. To investigate the changes in the expression levels of these nine conidiation-related genes in the Δ*Plhffp* and Δ*Plpif1* mutants, RT-qPCR was conducted to measure the expression levels of the nine genes, aiming to find the potential connection between the two deleted genes and these nine conidiation-related regulatory genes.

**FIGURE 7:**
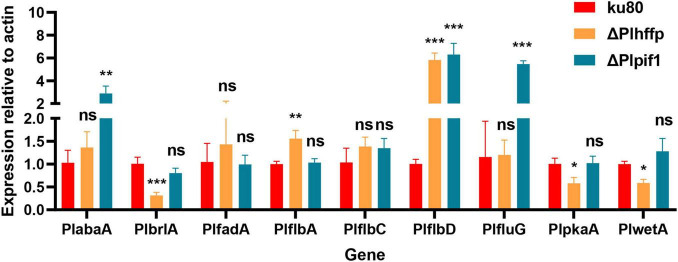
The expression levels of previously reported conidiation-regulating genes in *ku80* and the two mutants. *, *p* < 0.05; **, *p* < 0.01; ***, *p* < 0.001; ns, not statistically significant.

According to the experimental results (Figure 7), in Δ*Plhffp* mutant, the expression level of *PlbrlA*, which mainly regulates the production of conidia in *P. lavendulum*, was significantly decreased compared to the wildtype strain. In contrast, the expression level of *PlflbD* was significantly increased in Δ*Plhffp* mutant, consistent with its role of a negative regulator for conidiation in *P. lavendulum*. It is speculated that the *Plhffp* gene regulates conidiation in *P. lavendulum* through coordinated regulation of *PlbrlA* and *PlflbD* genes. Similarly, in the Δ*Plpif1* mutant, the expression level of *PlflbD* also significantly increased, consistent with its inhibitory effect on conidiation in *P. lavendulum*.

### 3.7 The two conidiation-regulating genes do not directly affect the nematocidal activity of the fungus

The nematocidal activities of the Δ*Plhffp* and Δ*Plpif1* mutants were measured and compared with the original strain *ku80*. Interestingly, there was no statistically significant difference in insecticidal activity between these two mutants and strain *ku80* (Supplementary Figure 6). However, considering that the experiments were conducted with an equal number of conidia, while the actual conidiation yields of Δ*Plhffp* and Δ*Plpif1* mutants were significantly lower than that of *ku80*, it can be concluded that the deletion of the *Plhffp* and *Plpif1* genes indirectly reduces the nematocidal activity of *P. lavendulum*.

## 4 Discussion

*Purpureocillium lavendulum* is an important nematode biocontrol fungus with the potential to be developed into a biological nematicide. The production and regulation of conidia (asexual spores) are crucial for the nematocidal activity of *P. lavendulum*. In this study, to investigate the regulatory mechanisms associated with conidial production in *P. lavendulum*, we constructed a T-DNA mutant library. Through screening the mutant library, we successfully identified two genes involved in the regulation of conidial production in this fungus. Gene knockout and complementation experiments confirmed their involvement in conidial production.

To construct a large number of T-DNA random insertion mutants, the fungus *P. lavendulum ku80* strain was transformed using ATMT. The initial screening of these mutants focused on their colony morphology to identify those with potentially reduced conidiation. Out of 2650 mutants screened initially, 131 exhibited abnormal conidiation, characterized by increased white mycelium production. Based on this result, we estimated that ∼5% of the genes in the entire genome of *P. lavendulum* may potentially affect conidia production. After the initial screening, two strategies, namely, second-generation sequencing and genome walking, were employed to identify the T-DNA insertion sites. These two approaches have been proven effective in other studies (Cottage et al., 2001; Chen et al., 2021; Edwards et al., 2022).

One mutant strain, #5-197, displayed white colonies, which remained unchanged even after several months of cultivation in test tubes, indicating low conidiation. The T-DNA insert site identified through whole-genome second-generation sequencing and confirmed by PCR, was predicted to disrupt an α, β-hydrolase-fold family protein, *Plhffp*. The α, β-hydrolase fold superfamily is an ancient and diverse group of major hydrolytic enzymes. The core structure of α, β-hydrolases consists of β-folds surrounded by α-helices (Wullich et al., 2020). These proteins are commonly found in bacteria and fungi. For example, 105 α, β-hydrolases have been identified in the human pathogenic bacterium *Mycobacterium tuberculosis* and many are involved in lipid metabolism, particularly in the biosynthesis and maintenance of its unique cell membrane. α, β-hydrolase fold proteins in *M. tuberculosis* also participate in immune evasion, immune regulation, detoxification, and metabolic adaptation. They play roles in growth, response to intracellular acidification, and dormancy (Johnson, 2017).

The growth of Δ*Plhffp* mutant on SDS-containing medium was retarded, suggesting that the deletion of the *Plhffp* gene may partially affect the integrity of the cell wall. Lipids are minor components of fungal cell walls and, in some cases, can participate in cell signaling (Beccaccioli et al., 2019). The homologous function of *Plhffp* is mainly associated with lipid metabolism (Lord et al., 2013), so it is speculated that the deletion of the *Plhffp* gene may affect the normal metabolism of some lipids in the cell wall, disrupting the integrity of the cell wall and reducing its ability to resist adverse environments. This is also evident in oxidative culture medium: when the concentration of H_2_O_2_ was 1 mM or 2.5 mM, the growth rate of Δ*Plhffp* showed no difference compared to the control, but when the H_2_O_2_ concentration increased to 5 mM, the growth of Δ*Plhffp* was significantly inhibited, and some plates did not produce any visible colonies. At H_2_O_2_ concentrations of 10 mM and 15 mM, Δ*Plhffp* failed to germinate and grow. In terms of conidiation, at concentrations of 1 mM, 2.5 mM, and 5 mM, the conidiation of Δ*Plhffp* was significantly lower than that of *ku80*. This indicates that the deletion of this gene severely affects the strain’s antioxidant capacity. Fungal cell walls are composed of complex components that may contain antioxidant substances such as glutathione, creating a suitable redox environment for the fungus. Therefore, it is hypothesized that the deletion of the *Plhffp* gene not only affects cell wall lipid metabolism but also reduces the content of antioxidant components in the cell wall, leading to a decreased ability to resist high oxidative conditions.

On a high-salt (NaCl) culture medium, the growth rate of Δ*Plhffp* was similar to *ku80*, indicating that the deletion of the *Plhffp* gene did not affect the strain’s ability to tolerate high osmolarity. In terms of conidiation, there was no significant difference in spore production between Δ*Plhffp* mutant and *ku80* on 0.1 M and 0.2 M NaCl media. Considering the significantly lower conidiation of the Δ*Plhffp* mutant compared to *ku80* on regular culture medium, it is speculated that the presence of NaCl creates a relatively unfavorable environment that triggers a response in Δ*Plhffp* mutant to counteract the adverse conditions. Conidiation is a fungal strategy to cope with unfavorable environments, and the increased conidiation in Δ*Plhffp* mutant may be a result of this response. As the NaCl concentration reached 0.3 M, there was no significant difference in colony size compared to *ku80*, but the final colony diameter of Δ*Plhffp* mutant was smaller, and the conidiation was significantly reduced compared to *ku80*. This suggests that the 0.3 M NaCl concentration has exceeded the threshold that the knockout strain can tolerate, thus affecting both colony growth and conidiation to some extent.

The results of RT-qPCR showed that in the Δ*Plhffp* mutant, the expression levels of three genes, PlbrlA, PlpkaA, and PlwetA, were significantly downregulated. The absence of *PlbrlA* inhibits conidial formation (Chen et al., 2020), *PlpkaA* is a suppressor gene for conidial production in *P. lavendulum* and other fungi (Baltussen et al., 2019), and *PlwetA* regulates conidial detachment, cell wall thickening, and other phenotypes related to conidial maturation (Etxebeste et al., 2010). The significant decrease in the expression levels of these three genes overall inhibits the production of conidia in *P. lavendulum*. On the other hand, the expression levels of two genes, *PlflbA* and *PlflbD*, were significantly upregulated. *FlbA* is an important upstream activating gene for conidial formation in *Aspergillus nidulans*, but its deletion in *P. lavendulum* does not affect conidiation (Chen et al., 2020). Further research is needed to understand its role in *P. lavendulum*. PlflbD suppresses conidial production in *P. lavendulum*. The significant upregulation of these two genes inhibits conidial production in *P. lavendulum*. The expression levels of *PlfadA* and *PlflbC* showed a slight increase but were not statistically significant. Both genes are suppressor genes for conidial production in *P. lavendulum*, and their partially increased expression levels have an inhibitory effect on conidial production.

After validating the results of genome walking, the T-DNA insertion site of mutant #5-119 was determined as an ATP-dependent DNA helicase. In wheat, knocking out the ATP-dependent DNA helicase gene (TaDHL-7B) using the CRISPR/Cas9 system resulted in reduced plant height and increased lodging resistance, while the yield remained unchanged (Guo et al., 2022). In the fission yeast, the ATP-dependent DNA helicase CHL1 can unfold DNA:RNA and DNA:DNA substrates *in vitro*. CHL1 plays an important role in maintaining genome stability by participating in DNA repair and sister chromatid cohesion (He et al., 2022). In eukaryotes, the Pif1 helicase family has multiple roles in maintaining nuclear DNA and mitochondrial DNA integrity. The ScPif1 (*S. cerevisiae Pif1*) helicase in the budding yeast *Saccharomyces cerevisiae* is involved in replication by participating in replication barriers such as G-quadruplexes and protein blocks, reducing genetic instability at these sites. *ScPif1* also regulates telomerase, promotes Okazaki fragment processing, and works with polymerase δ in break-induced repair (Geronimo and Zakian, 2016). Another Pif1 helicase in the budding yeast, Rrm3, assists fork progression at replication fork barriers on rDNA loci and tRNA genes. (Byrd and Raney, 2017). In both budding and fission yeasts, the Pif1 helicase family affects both the semiconservative replication of telomeric DNA mediated by telomerase and the recombinational elongation of telomeres (Geronimo and Zakian, 2016).

The growth rate of Δ*Plpif1* mutant was not significantly different from *ku80* and the complementation strain on MM, TG, and CMA media, indicating that the deletion of the *Plpif1* gene does not affect the normal growth of the strain. The deletion of the *Plpif1* gene resulted in significantly decreased conidiation in all the three media. Complementation of the deletion mutant rescued that phenotype. On a culture medium containing NaCl, there were no significant differences in colony morphology, growth rate, or conidiation between the Δ*Plpif1*, Δ*Plpif1-C* and *ku80* strain. This indicates that the knockout of the *Plpif1* gene does not affect the strain’s ability to regulate osmotic pressure. On a medium containing SDS, the colony size and growth curve of the Δ*Plpif1* strain were not significantly different from the *ku80* strain, suggesting that the deletion of this gene does not affect the cell wall integrity of the strain.

On culture media with low concentration (1 mM) and median concentration (2.5 mM) of H_2_O_2_, the final colony diameter of the knockout strain was comparable to the control, but on a medium with a high concentration (5 mM) of H_2_O_2_, the colony diameter of the knockout strain was significantly smaller than the control. This indicates that the knockout strain has weaker antioxidant capacity. The conidiation of the knockout strain was similar to the control on 1 mM H_2_O_2_ medium but significantly lower than the control on 2.5 mM and 5m M H_2_O_2_ media. Overall, knockout of the *Plpif1* gene mainly affects the strain’s conidiation but not growth and stress tolerance.

The results of RT-qPCR showed that the expression levels of *PlbrlA* were downregulated although the change was statistically not significant. The negative regulator PlflbD was found upregulated. Based on this, we hypothesize that Plpif1 may act on PlflbD, thereby inhibiting the expression of *PlbrlA* and suppressing conidiation. The specific interactions between these two genes require further investigations.

The above study identified two new genes involved in conidiation regulation in *P. lavendulum*, *Plhffp* and *Plpif1*. However, the regulatory mechanisms involving these two genes require further investigation. To comprehensively address conidiation regulation, more conidiation-deficient mutants in our library need to be analyzed.

## Data availability statement

The datasets presented in this study can be found in online repositories. The names of the repository/repositories and accession number(s) can be found below: NCBI – PRJNA1066049.

## Ethics statement

The manuscript presents research on animals that do not require ethical approval for their study.

## Author contributions

YW: Data curation, Investigation, Methodology, Writing—original draft. F-NQ: Investigation, Methodology, Writing—review and editing. Y-RX: Investigation, Methodology, Writing—review and editing. K-QZ: Funding acquisition, Supervision, Writing—review and editing. JX: Writing—review and editing. Y-RC: Funding acquisition, Methodology, Writing—review and editing. L-ML: Conceptualization, Funding acquisition, Investigation, Methodology, Project administration, Supervision, Validation, Writing—original draft, Writing—review and editing.
