# Supplementary material for: Characterization of regulatory genes Plhffp and Plpif1 involved in conidiation regulation in Purpureocillium lavendulum
Source: Front Microbiol. 2024 Feb 15;15:1352989. doi: 10.3389/fmicb.2024.1352989 (PMC10906660; doi:10.3389/fmicb.2024.1352989)
Supplement: Supplementary file 1 [file Data_Sheet_1.pdf]

Supplementary Materials

Characterization of Regulatory Genes *Plhffp* and *Plpif1* Involved in Conidiation Regulation in *Purpureocillium lavendulum*

Yu Wei[1], Feng-Na Qi[1], Yan-Rui Xu, Ke-Qin Zhang[1], Yan-Ru Cao[2]*, Lian-Ming Liang[1]*

[1] State Key Laboratory for Conservation and Utilization of Bio-Resources in Yunnan and The Key Laboratory for Southwest Microbial Diversity of the Ministry of Education, Yunnan University, Kunming, China.
[2] College of Agriculture and Life Sciences, Kunming University, Kunming, PR China.

*Correspondence: lianglm@ynu.edu.cn (L-ML); yanrucao3@aliyun.com(Y-RC)

Fig. S1 Fungal strain preservation evaluation. (A) Germination status of strains after 1-month preservation. (B) Germination status of strains after 6-month preservation.

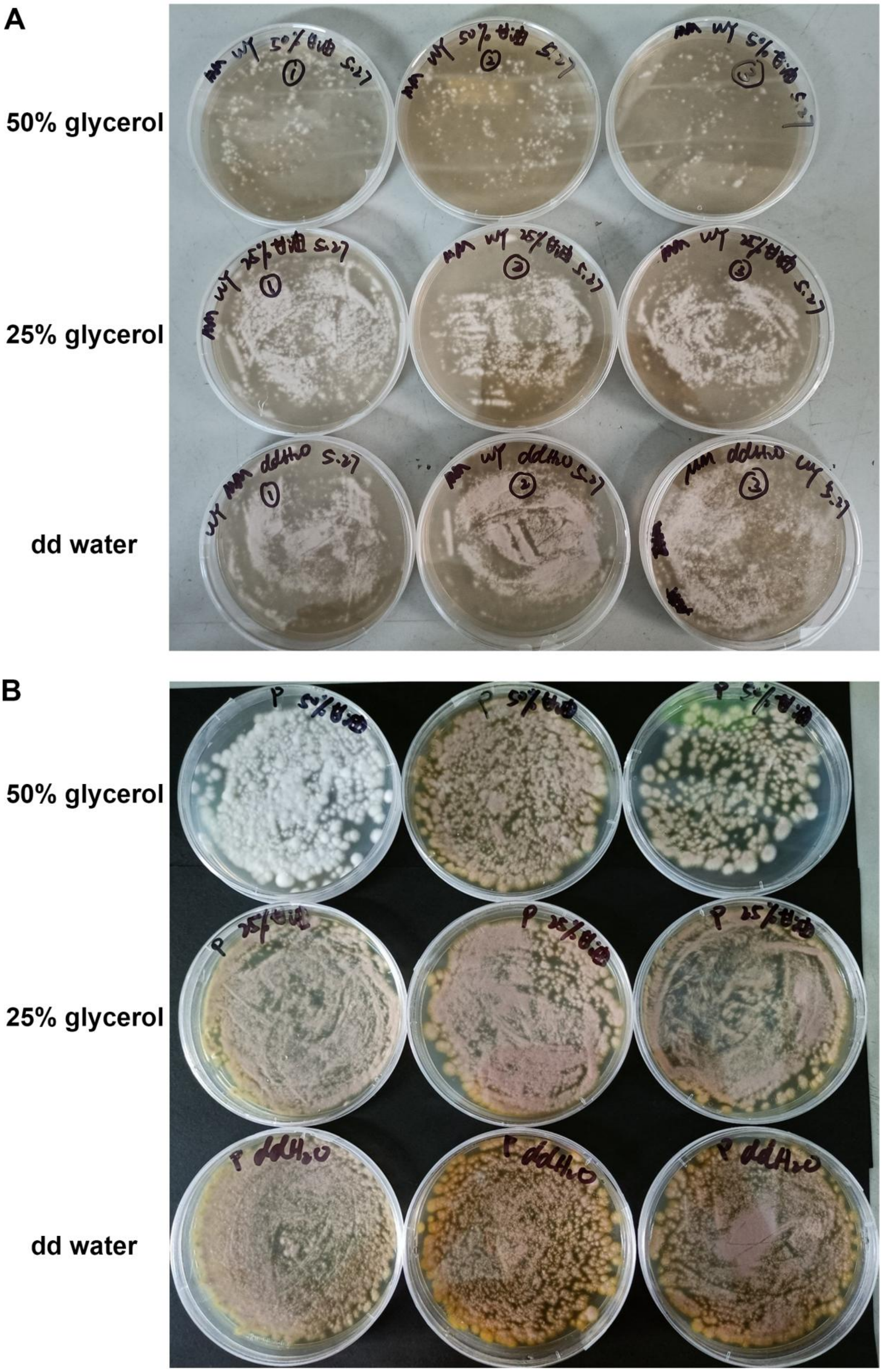

Fig. S2 The growth curve of ku80 and *Plhffp* mutants on different media.

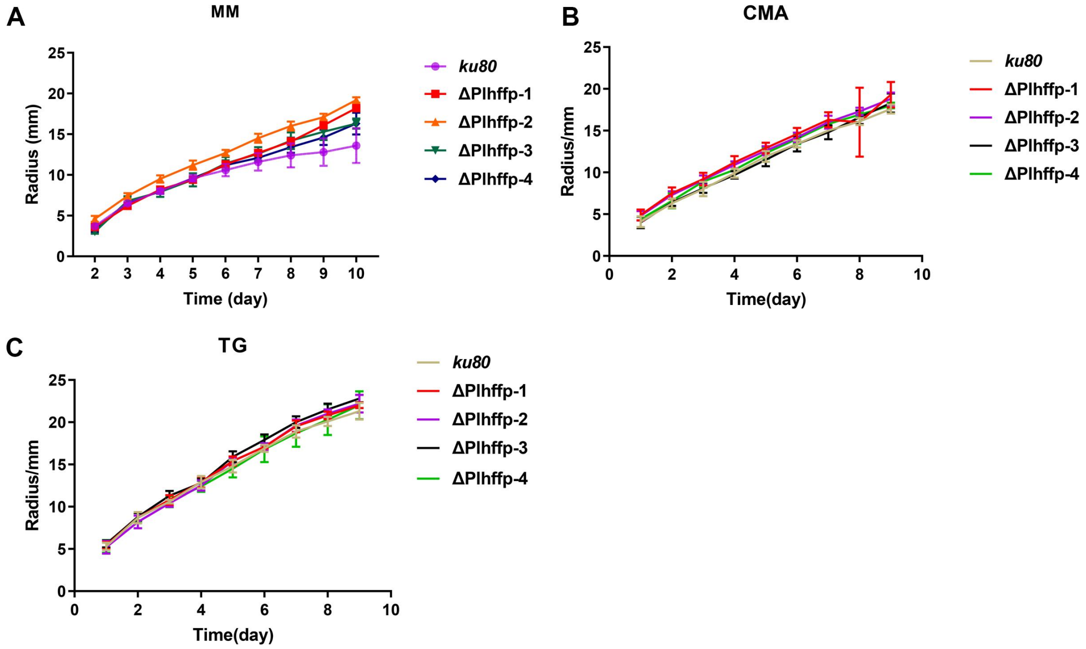

Fig. S3 The growth and conidiation of ku80 and *Plhffp* mutants on high osmotic media. *, $p<0.05$; **, $p<0.01$; ***, $p<0.001$; ns, not statistically significant.

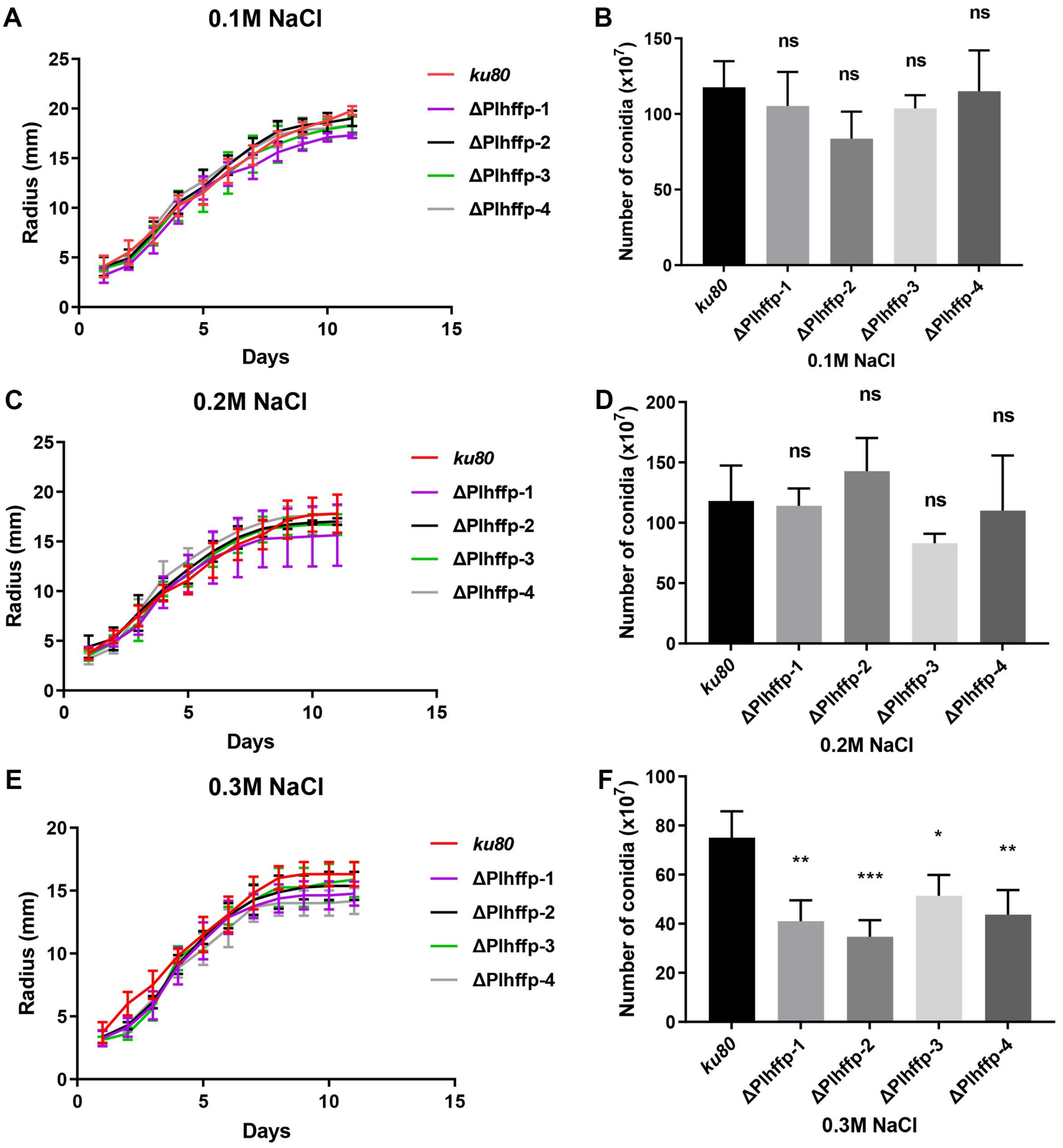

Fig.S4 Comparison of growth and conidiation of ku80 and the Δ*Plhffp* strains on SDS containing media. *, p<0.05; **, p<0.01; ns, not statistically significant.

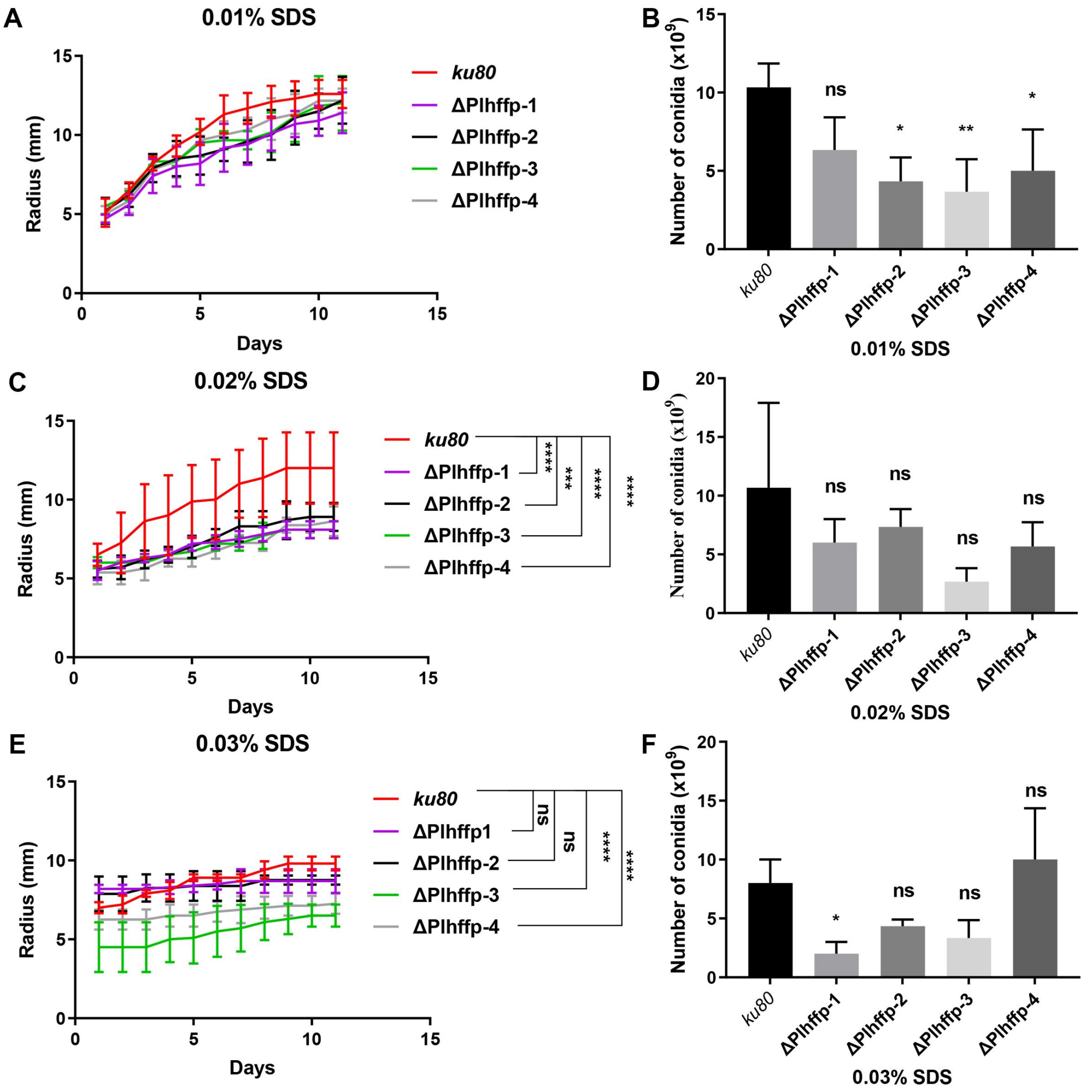

Fig.S5 Comparison of growth and conidiation of ku80 and the Δ*Plhffp* strains on H_2O_2 containing media. *, p<0.05; **, p<0.01; ***, p<0.001; ns, not statistically significant.

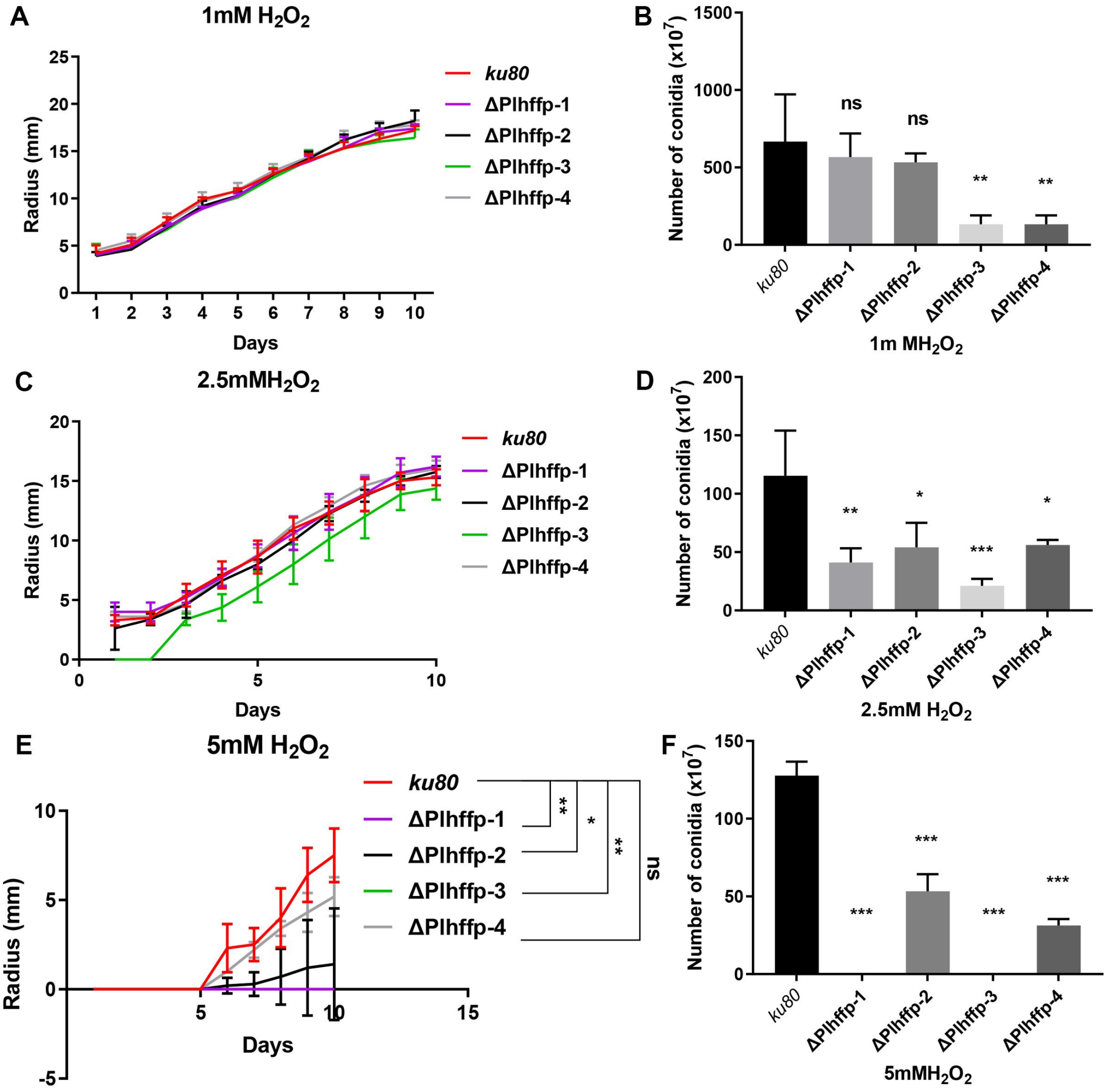

Fig. S6 the bio-assay of ku80 and the two mutants against *C. elegans*.

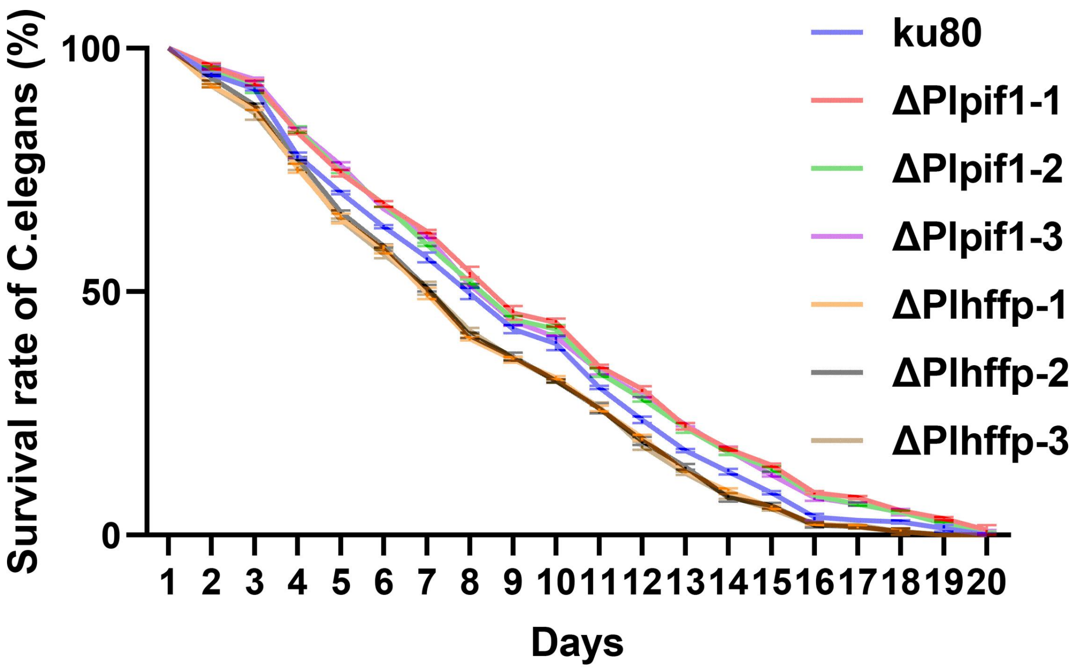

Table S1 Primers used in this study

Primer name	Primer sequence	Amplification purpose
surF<br>surR	TCATCCAAGGGATGTTCACG<br>AACAAAGTCGATGTGCTTCG	Amplify the *sur* gene fragment.
5-197C1X<br>A F	CCTTTGAGCCGTGTGTTCG<br>CAACAGTACGAAGCATTTCG	Validate the insertion site of the T-DNA in the 5-197 mutant strain.
SP1	CCAGCCGGGAGATGTGGGGC	Gene Walking
SP2	AACGTCGTGACTGGGAAAACCCT	Gene Walking
SP3	CCCTTCCCAACAGTTGCGCA	Gene Walking
1577 3F<br>1577 3R	CACGAGGACTTCTAGCCAGCACAATCCACAGCA<br>GCGTTGGCACAGATCCCGTTACGAATGAACCTCTCCC	Amplify the upstream homologous arm of the *Plhffp* gene (underlined sequence indicates the homologous arm for infusion cloning).
1577 5F<br>1577 5R	ATGCTCTCACACTAGCCACCTCCTCTCTTCTCCCT<br>GACGGAATTGAGGATAACACCTACGCTATCTATTGAC<br>G	Amplify the downstream homologous arm of the *Plhffp* gene (underlined sequence indicates the homologous arm for infusion cloning).
1577 inner F<br>1577 inner R	TCTTCTCGATGTCCGTCG<br>ATGGACTGACAAGCACA	Amplify replaced fragment of *Plhffp* gene in gene knockout screening.
tubulin F<br>tubulin R	GCTCAGCACCCTCAGTGTAGT<br>TGGGATTTTTGTTCGTCGTAT	Amplify tubulin fragment.
PIF1 3F<br>PIF1 3R	CACGAGGACTTCTAGAGCCTGGTCCTTGGGTTGGT<br>GCGTTGGCACAGATCTGGTTTCTCCTCGCCACAGC	Amplify the upstream homologous arm of the *Plpif1* gene (underlined sequence indicates the homologous arm for

		infusion cloning).
PIF1 5F<br>PIF1 5R	ATGCTCTCACACTAGTGGCGATAGCGTGACTTG<br>GACGGAATTGAGGATGCACCGATTACGGAACCTC	Amplify the downstream homologous arm of the *Plpif1* gene (underlined sequence indicates the homologous arm for infusion cloning).
c9 inner F<br>c9 inner R	TGCGCACAGATGAGATTCAAGTCAGAG<br>CACCAGCATGATGTTCACATGAA	Amplify replaced fragment of *Plpif1* gene in gene knockout screening.
PIF1 hb F<br>PIF1 hb R	ACTGCTGGCCTCTAGTTGCGATGGCACAATTTGCAAA<br>AAACACTGATAGTTTTTCCCAGAAGGACAAAGTCAG<br>G	Amplify the whole gene of *Plpif1 for comlementation* ((underlined sequence indicates the homologous arm for infusion cloning).
RT-actin-F<br>RT-actin-R	GAGGTAGTCGGTCAAGTCGC<br>TCCCATCAACCCCAAGTCC	Real time PCR primers for actin.
RT-abaA-F<br>RT-abaA-R	AGCTTGGGAGGAGTTGAGGA<br>TTAGGCAGTACCGCAACAGG	Real time PCR primers for *PlabaA*.
RT-brlA-F<br>RT-brlA-R	AGAAGGAAATGGGGCTGTCG<br>TTCCGGTCCCTATCCCATGT	Real time PCR primers for *PlbrlA*.
RT-fadA-F<br>RT-fadA-R	CGCGTGCGACTACATCCTTA<br>TTCAAGAGCCAGCCAGTCAG	Real time PCR primers for *PlfadA*.
RT-flbA-F<br>RT-flbA-R	GCTCCGTAACCTGCTTCTGT<br>CTATTTGCCAGCGCTTCGTC	Real time PCR primers for *PlflbA*.
RT-flbC-F<br>RT-flbC-R	GAAGCTCCTCCGGTCATCTG<br>GTCGCTACTCCCATCACCAG	Real time PCR primers for *PlflbC*.
RT-fluG-F<br>RT-fluG-R	AAGGGCGTCGTGGGCAATA<br>CGGTCGTGGAGACATCGTG	Real time PCR primers for *PlfluG*.
RT-pkaA-F	TCCAGTCCTGAAATGTGCCC	Real time PCR primers for

RT-pkaA-R	CTCAAGAAGGCCCAAGTCGT	*PlpkaA*.
RT-flbD-F<br>RT-flbD-R	CCATCACCATCCTCGTCCTG<br>CTGCCTCAACATCATCGGGT	Real time PCR primers for<br>*PlflbD*.
RT-wetA-F<br>RT-wetA-R	ACCTGTCCCAGCAATTCGAG<br>GCGCCTCGACAAACTTCTTG	Real time PCR primers for<br>*PlwetA*.
